# Impact of smoking intensities on sleep quality in young Saudi males: a comparative study

**DOI:** 10.25122/jml-2022-0216

**Published:** 2022-11

**Authors:** Arwa Al-Mshari, Mona Hmoud AlSheikh, Rabia Latif, Sadaf Mumtaz, Waleed Albaker, Mohammed Al-Hariri

**Affiliations:** 1 Department of Biomedical Sciences, College of Medicine, King Faisal University, Hofuf, Saudi Arabia; 2 Physiology Department, College of Medicine, Imam Abdulrahman Bin Faisal University, Dammam, Saudi Arabia; 3 Physiology Department, Dental College, HITEC-Institute of Medical Sciences, Taxila, Pakistan; 4 Department of Internal Medicine, College of Medicine, Imam Abdulrahman Bin Faisal, Dammam, Saudi Arabi

**Keywords:** sleep quality, smoking index, Pittsburgh Sleep Quality Index, young males, insomnia

## Abstract

This study aimed to compare various components of sleep quality between cigarette smokers of various intensities and non-smokers in young Saudi males. In total, 73 healthy male participants (31 smokers and 42 non-smokers) aged 17–33 years were recruited over three months (August 2018 to October 2018). All participants completed the Pittsburgh Sleep Quality Index (PSQI) questionnaire. The smokers were then divided into three groups, according to their Smoking Index* (SI) (Cigarettes Per Day (CPD) X Years of Tobacco Use), into mild, moderate, and heavy smokers. The PSQI was significantly higher in heavy smokers than in mild smokers (P=0.022) or non-smokers (p=0.013). A significant positive correlation was observed between the PSQI and the smoking index (p=0.005). Sleep duration was significantly longer in heavy smokers compared to mild (p=0.032) and nonsmokers (p=0.047). Sleep disturbance was significantly higher in moderate than nonsmokers (p=0.035) and moderate than mild smokers (p=0.028). Sleep latency was significantly longer in heavy than nonsmokers (p=0.011). Daytime dysfunction was significantly higher in moderate than mild smokers (p=0.041). Habitual sleep efficiency was significantly greater in moderate than in either mild (p=0.013) or nonsmokers (p=0.021). The use of sleep medication was significantly higher in moderate than nonsmokers (p=0.041). The findings suggest that poorer sleep quality is positively associated with smoking intensity among young Saudi males. Considering the importance of sleep quality for well-being and health, these results suggest exploring how improving sleep quality could inform future smoking cessation interventions.

## INTRODUCTION

Tobacco smoke has many hazardous compounds known to be highly toxic to the brain and nervous system [1]. Smoking may induce cognitive impairment either directly through exposure to these substances [2–4] or indirectly by impairing other body systems, such as the cardiovascular [5, 6] and respiratory [7, 8] systems. Sleep is a dynamic physiological phenomenon essential for maintaining normal mental health and mood. During sleep, many psychological tensions and neurological derangements are healed. Sleep disturbances and poverty of sleep quality predispose individuals to emotional instability, mood disturbances, and mental illness [5]. Moreover, lack of quality sleep has physical consequences as it also affects cardiovascular, respiratory, immune, and endocrine functions [6]. Importantly, smoking can affect health through accelerated epigenetic aging (DNAm-based measure of biological aging) with the development of diseases and earlier death in later life [9].

Previous studies consistently found that smokers report subjectively poorer sleep than non-smokers [10–14]. In addition, recent data have shown that smokers have less total sleep time, lower sleep efficiency, and a longer latency to sleep onset [15, 16]. While fewer objective studies have been conducted, Zhang et al. [17] used electrophysiologic recordings of eye, muscle, and brain waves to examine the changes in duration, frequency, and latency of various sleep stages between smokers and non-smokers.

This study showed that the sleep cycle was disrupted with lighter stages predominating. In addition, the ponto-geniculo-occipital (PGO) spikes responsible for dreams were affected, and the rapid eye movement (REM) sleep was delayed and of shorter duration.

Nicotine, the active ingredient in tobacco, is a neurotransmitter that acts on nicotinic acetylcholine receptors in the brain [9]. These receptors are widely distributed in the brain [10]. Activation of nicotinic receptors leads to the release of several other neurotransmitters [11].

Investigations conducted *in vivo* demonstrated the disruption of the ponto-geniculo-occipital (PGO) spikes and the electrophysiologic basis of the dream by nicotine. This effect is dose-dependent and could be due to stimulation of the serotonergic neurons [18].

To the best of our knowledge, it is not known whether smoking disrupted sleep or whether people with poor sleep resorted to smoking among young males in Saudi Arabia. Moreover, smoking is more common in males than females in Saudi Arabia. Thus, our study investigated this relationship by trying to find a dose-dependent influence between smoking intensities and sleep quality in young Saudi males.

## MATERIAL AND METHODS

All participants were recruited using purposive sampling (non-probability sampling) over three months between August 2018 and October 2018. Individuals were recruited through family members, the community from the medical center of Imam Abdurrahman bin Faisal University, and the quit smoking clinic in Dammam, Saudi Arabia. The inclusion criteria were healthy males 17–33 years of age, who were either current cigarette smokers of at least one cigarette/day for six months or more or non-smokers who had never smoked tobacco in their life or were exposed only as secondary smokers. The exclusion criteria were former smokers, subjects with a genetic or organic mental disorder, psychiatric disorder, skeletal muscle disorder, sleep disorder, sedative or hypnotic intake, asthma, sickle cell disease, alcoholics, individuals who smoked other substances, such as cannabis, cocaine, or heroin, and subjects under pharmacological or behavioral smoking cessation programs.

The study employed the Smoking Index (SI), which is calculated by multiplying the cigarettes smoked per day by the number of years of smoking [SI=Cigarettes Per Day (CPD) X Years of Tobacco Use].

The Pittsburgh Sleep Quality Index (PSQI) instrument, which was used to measure perceived sleep quality and duration over a one-month period, consists of 19 subjective questions which evaluate sleep by producing seven component scores: sleep duration, sleep latency, habitual sleep efficiency, subjective sleep quality, use of sleep medications, sleep disturbances, and daytime dysfunction.

The final PSQI score was determined by totaling the seven components' scores to obtain a global score ranging from 0 to 21, with higher scores denoting poorer sleep quality. The original authors of the PSQI established a cut-off of >5 for the global score to distinguish between poor sleepers and good sleepers (<5) [19]. This study used the Arabic version of the PSQI, which has been tested and demonstrated good reliability and validity [20, 21].

### Data analysis procedure

The Statistical Package for the Social Sciences (SPSS) (version 1.0.0.1162) was used to conduct the data analysis. Testing for normality of distribution was performed using the Shapiro-Wilk test. Normally distributed data are presented as mean and standard deviation (SD), whereas non-normally distributed data are presented as median and interquartile range (IQ)R. A comparison between the two groups (smokers and non-smokers) was conducted using an unpaired student's t-test for the parametric data and the Mann-Whitney test for the non-parametric data. The Chi-Square test (χ2) was used for the qualitative data. Pearson's correlation was used for the parametric data, and Spearman's correlation was used for the non-parametric data. Mean values and standard deviations were used for the normally distributed data. Median values with an interquartile range were used for the non-normally distributed data. A p-value of <0.05 was taken as statistically significant.

## RESULTS

Of the 73 participants, 42.5% (n=31) were smokers, and 57.5% (n=42) were non-smokers. The two groups differed significantly in age, marital status, and monthly income. The non-smokers were significantly younger (p<0.001), more likely to be unmarried (single) (p<0.001), and had a significantly lower monthly income (p=0.04) than the smokers, as presented in Table 1.

**Table 1:** Basic characteristics of participants.

		Smokers (n=31)	Non-smokers (n=42)	P-value
**Age (years)**	Median (IQR)	24 (7)	22 (5)	<0.001
**Marital status**	Unmarried	23 (74.2%)	42 (100%)	0.001
	Married	8 (25.8%)	0	
**Monthly income (Saudi riyals)**	<5,000	16 (53.3%)	33 (78.6%)	0.044
	5,000–10,000	11 (36.7%)	7 (16.7%)	
	>10,000	3 (10%)	2 (4.7%)	

IQR – interquartile range.

The PSQI score of smokers was 6.1±3.4, and 4.9±2.2 among non-smokers. Since the unpaired student t-test showed no significant difference in the total sleep scores between the two groups (p=0.145), the Pearson correlation test was performed between SI and PSQI, which found that within the smoker group, there was a significant positive correlation (p<0.001) between the SI and the PSQI, indicating that heavy smoking was correlated with a poorer quality of sleep. Meanwhile, the overall PSQI showed significantly higher values, or a poorer quality of sleep, for heavy smokers than the mild or non-smokers (Table 2). The trend showed a progressive increase in most sleep components between the non-smoker, mild smoker, moderate smoker, and heavy smoker groups, indicating a stepwise worsening of sleep quality.

**Table 2:** Correlation of Smoking Index with Pittsburgh Sleep Quality Index among the study groups.

		Smokers			
		Non-smoker	Mild	Moderate	Heavy
Number of responses		**40**	**14**	**5**	**2**
**PSQI**	Median	4	4	9	10.5
	IQR	3	4	5	3
**P-value**	Overall p-value*	0.008			
	Heavy versus non-smokers**	0.009			
	Heavy versus mild smokers**	0.033			
**Correlation (r)**	PSQI and smoking Index***	0.598 (p=0.004)			

IQR – interquartile range; * – Kruskal Wallis Test; ** – Mann-Whitney U test; *** – Spearman's Rho.

Table 3 presented the pairwise comparison of the various components of the SI between different non-smoker and smoker groups and showed a significant difference in the following categories: sleep duration (significantly longer in heavy smokers than in non-smokers and mild smokers), sleep disturbance (significantly greater in moderate smokers than in non-smokers and in mild smokers), sleep latency (significantly longer in heavy smokers than in non-smokers), daytime dysfunction (significantly greater in moderate than in mild smokers), habitual sleep efficiency (significantly greater in moderate than in mild smokers and non-smokers), and sleep medication which was used significantly more by the moderate smokers than non-smokers.

**Table 3:** Pairwise comparisons of the mean PSQI, according to smoking groups.

Components	Smokers				P-value
	Non-smoker	Mild	Moderate	Heavy	
	Mean±SD				
**Sleep duration**	0.68±0.92	0.5±0.76	1±1	2±1.41 ^a, b^	0.155
**Sleep disturbance**	0.93±0.35	0.86±0.66	1.4±0.55 ^a, b^	1.5±0.71	0.053
**Sleep latency**	0.9±0.78	1.36±1.01	1.2±0.84	2.5±0.71 ^a^	0.035
**Daytime dysfunction**	1.05±0.68	0.79±0.89	1.6±0.89 ^b^	1.5±0.71	0.173
**Habitual sleep efficiency**	0.5±0.93	0.29±0.83	1.6±1.34 ^a, b^	1.5±2.12	0.042
**Subjective sleep quality**	0.68±0.53	1±1.24	1.2±1.1	1.5±2.12	0.254
**Use of sleep medication**	0.1±0.38	0.14±0.36	0.6±1.34 ^b^	0±0	0.22

SD – Standard deviation; PSQI – Pittsburgh sleep quality index; a>Non-smoker; b>Mild smokers.

## DISCUSSION

While countless previous studies demonstrated the deleterious effects of smoking and its impact on quality of life, the effect of smoking on sleep is controversial in terms of whether nicotine or tar disrupts the physiology of the sleep cycle, reducing the duration of deep slow-wave sleep (SWS) stages and REM stage in particular, and whether subjects with insomnia resort to smoking to alleviate their anxiety and obtain momentary relaxation. Previous studies reported a positive association between smoking and sleep disorders, insomnia, or poor sleep quality [9–18, 22]. However, they differed in their conclusion regarding the cause of this association, with some attributing it to breathing problems and sleep apnea, which is directly worsened by smoking [2, 4, 5, 9, 12], while others suggested that the sleep disruption was caused indirectly by the worsening of chronic rheumatic conditions [7, 11, 12, 16, 23]. Meanwhile, a recent review paper concluded the direct influence of nicotine on the sleep/wake cycle [24], and the objective polysomnography study conducted by Zhang and Jaehne et al. [16, 17] demonstrated that the neurophysiological mechanism of PGO spikes during REM sleep was disrupted by nicotine.

The results of the present study showed a positive correlation between the frequency of smoking and poverty of sleep quality among young Saudi males, supporting the view of the direct effect of nicotine on the central nervous system, affecting the mechanism of the sleep-wake cycle and the circadian rhythm [16, 17]. Nicotine is a neurotransmitter and acts on the nicotinic acetylcholine receptors in the brain, engendering these sleep changes [13]. Nicotine is known to alter the monoaminergic neurotransmitter balance in the brain [22]. This study found that the overall PSQI showed a significantly higher value for heavy smokers than non-smokers, indicating poorer sleep quality in the former.

Moreover, the analysis of the SI, as measured by the PSQI, showed a clear trend of worsening sleep quality between the non-smoker group and the various smoker groups in a stepwise fashion, while the results of the significant pair comparison demonstrated the involvement of all sleep components, except the overall subjective sleep quality. The total Sleep Quality Index for all the groups was less than three, indicating that the sleep quality of the sample was poor but did not require medication. This finding can be explained by the nature of the sample, which involved a young age group and a relatively short duration of smoking. The study was designed to include young male adults to avoid the influence of the cyclic hormonal changes of female subjects and the older age co-morbidities, such as sleep apnea and arthritis.

The increase in sleep duration among smokers can be related to the influence of nicotine on REM sleep or to the fact that it is a compensatory mechanism for low sleep quality. Meanwhile, the increase in sleep disturbance was expected because the stimulant nature of nicotine was unlikely to be related to worsening respiratory function or other inflammatory or connective tissue conditions, as these were part of the exclusion criteria, although subclinical conditions may have existed. The prolongation of sleep latency was related to the stimulant nature of nicotine on the acetylcholine nicotinic receptors in the reticular activating system, causing increased alertness and consciousness levels in smokers. These results supported the findings of previous studies [9–14]. The only difference was in the total sleep time component of the PSQI, as the present study found that the total sleep time for smokers was significantly higher than for non-smokers, in a dose-related fashion, contradicting most previous studies that reported a reduction of total sleep duration for smokers [9–14].

It is ironic that habitual smokers subjectively state that they need to smoke to help them relax and sleep since, physiologically, nicotine disrupts the sleep-wake cycle and the REM sleep PGO spike, the physiological basis for dreams. Nicotine also disrupts the mitochondria function by changing its enzymatic activity (increased lipid peroxidase), thus damaging mitochondrial DNA and increasing oxidative stress [24]. This causes a disruption of the circadian rhythm (smoking is well-known to decrease 5HT function and to cause the down-regulation of MAO) [22]. It also changes the levels of neurotrophic factors, such as BDNF [15, 25]. In addition, anxiety has an additive effect to nicotine on neurotransmitters [19].

The limitation of this study is that it was based on self-reported sleep quality. Excluding female and older subjects with comorbidity reduced the number of subjects. Larger studies of young individuals are required to investigate better the association between poor sleep quality and smoking intensity.

Objective studies are needed to determine the direct effect of smoking on the different phases of the sleep cycle. Furthermore, the present study suggested exploring how improving sleep quality could inform future smoking cessation interventions.

## CONCLUSION

The findings suggest that poorer sleep quality is positively associated with smoking intensity among young Saudi males.

## Data Availability

The data supporting these findings are available from the corresponding author upon reasonable request.
